# Bridging language gaps: a study of linguistic transfer perceptions among Chinese medical students in an English-medium instruction program

**DOI:** 10.3389/fmed.2026.1798909

**Published:** 2026-05-04

**Authors:** Jonathan Jenkin Tsui, Evelyn Yiyin Li, Cecilia Sicong Xie, Zhouyu Zhu, Xin Yin, Ban Chi-Ho Tsui, Alice Kwai-Yee Siu

**Affiliations:** 1 School of Medicine, The Chinese University of Hong Kong, Shenzhen, China; 2 Department of Anesthesiology, Perioperative and Pain Medicine, Stanford University, Stanford, CA, United States

**Keywords:** bilingual competence, China, English-medium instruction, language transfer, licensure examination, medical education, pilot study

## Abstract

**Background:**

English-Medium Instruction (EMI) in medical education aims to cultivate globally competent physicians, yet a significant challenge arises when students must subsequently pass high-stakes licensure examinations administered in their native language. This pilot study explores this critical linguistic transition by examining the post-examination perceptions of Chinese EMI medical students who completed the National Competency Test (NCT), a major Chinese-language benchmark examination.

**Methods:**

A cross-sectional survey was administered to 24 fourth-year EMI medical students following their NCT. Participants rated their confidence in performing parallel medical tasks in English and Mandarin (10 items each) and evaluated the perceived utility of EMI training for the Chinese clinical context (7 items). Data were analyzed using descriptive statistics, effect sizes (Cohen’s d), and paired *t*-tests to quantify language-based confidence gaps.

**Results:**

Despite achieving outstanding NCT results substantially above national averages, students reported a notable confidence gap favoring Mandarin for productive clinical communication skills, while receptive tasks showed minimal language differences. Students’ confidence in completing an Objective Structured Clinical Examination in Mandarin correlated positively with their actual examination performance. However, students did not attribute their examination success to their EMI training, revealing a disconnect between objective achievement and perceived utility.

**Conclusion:**

This study reveals a critical disconnect in EMI medical education: while students successfully transfer English-acquired knowledge to excel in a native-language licensing examination, they do not attribute this success to their EMI training and express low confidence in clinical English communication. These findings underscore the urgent need for EMI curricula to incorporate explicit pedagogical strategies that bridge English-acquired medical knowledge with native-language clinical application, thereby enhancing both perceived utility and readiness in linguistically diverse healthcare environments.

## 1 Introduction

There is widespread acknowledgement that the English-Medium Instruction (EMI) medical curriculum in China can encounter linguistic challenges: students acquire foundational knowledge in English while concurrently managing clinical rotations, professional assessments, and licensing examinations in Mandarin Chinese (1). Although it may seem challenging, engaging with medical concepts in two languages can provide cognitive and educational benefits. Access to the extensive collection of medical literature and resources in English enables a more profound engagement with the subject (2). Moreover, the imperative to understand and assimilate knowledge across linguistic boundaries can foster a more profound conceptual comprehension than mere rote memorization, as the process of learning and alternating between languages on the same topic necessitates an emphasis on fundamental principles (3, 4). This suggests that when employed effectively, EMI may not be a hindrance but instead promote a more robust and transferable knowledge base.

In China, medical education culminates in high-stakes licensure examinations that determine students’ eligibility to practice. The National Medical Licensing Examination (NMLE) serves as the definitive assessment for medical practice authorization, while the National Competency Test (NCT) functions as a standardized precursor examination administered in Mandarin Chinese (5, 6). The NCT functions as a standardized examination, consisting of a computer-based medical knowledge component and a multi-station clinical skills Objective Structured Clinical Examination (OSCE) to evaluate preparedness for clinical clerkships and forecast NMLE performance (7).

The Faculty of Medicine at The Chinese University of Hong Kong (CUHK) has historically complied with the registration standards of the United Kingdom General Medical Council (GMC) (8). This clinical medicine curriculum was then modified and adapted in 2021 for the new campus of the Chinese University of Hong Kong, Shenzhen (CUHK-Shenzhen), situated in China (9). Hong Kong possesses a historical legacy of English-language medical education, commencing with the founding of The University of Hong Kong in 1887. The founding of The Chinese University of Hong Kong (CUHK) in 1981 reinforced this tradition, establishing a bilingual academic and professional milieu. Consequently, the healthcare system in Hong Kong operates predominantly in English for academic assessment, medical documentation, and institutional communication (10). Proficient clinical practice requires fluency in Cantonese Chinese, the predominant language spoken at home by roughly 88.2% of the population (11). This necessitates that physicians trained in Hong Kong develop robust bilingual competencies to engage with patients and conduct history-taking in Cantonese, notwithstanding an English-dominated educational setting (12). In medical education, Cantonese and Mandarin, while classified as Chinese languages, lack mutual intelligibility, resulting in linguistic complexity (13).

**Figure 1:**
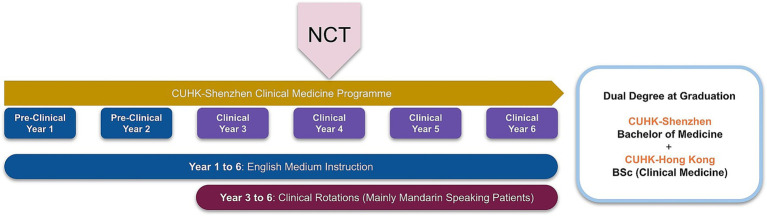
The 6-year clinical medicine program curriculum. Didactic instruction is delivered in English (years 1–6). Supervised clinical encounters, where patients primarily speak Mandarin Chinese, begin in year 3. (NCT: National Competency Test).

As mentioned above, the 6-year MBChB program at The Chinese University of Hong Kong (CUHK) was extended to its new Shenzhen campus in 2021. The overall curriculum arrangement is shown in Figure 1, implementing a Hong Kong-style EMI medical curriculum in mainland China. At this campus, all formal instruction is delivered in English; however, the local community and healthcare system cultivate a unique learning environment in contrast to Hong Kong. Census data indicate that roughly 66% of Shenzhen inhabitants use Mandarin Chinese, 31% utilize Cantonese Chinese, and only 24.2% assert functional proficiency in English (14). Census data reveals that 53.2% of Hong Kong’s populace is proficient in English (15). The percentage of English speakers is significantly greater than that in Shenzhen.

As a result, students obtain their medical knowledge in English but must utilize it in a primarily Mandarin-speaking clinical setting beginning in their third year. This denotes a crucial shift, in which knowledge and skills acquired in English must be recalled, applied, and communicated during Mandarin-language clinical practice and patient interactions.

The linguistic context here differs from established EMI medical programs (16), which often operate in regions with a historical English legacy or higher societal bilingualism such as South Asia and Hong Kong. This program introduces an EMI curriculum into a predominantly monolingual Mandarin environment, which may present unique implementation challenges due to students’ limited opportunities for an immersive English environment outside the classroom (17).

Existing research on EMI in medical education has focused on language proficiency and student satisfaction (18). However, there is less research examining student perceptions regarding the utility of EMI in taking native-language medical assessments. Understanding how students perceive the transferability of their EMI training after examination is crucial for evaluating the real-world effectiveness of these programs and for designing targeted support for native students (19).

This pilot study addresses this gap by investigating the immediate post-examination perceptions of Chinese EMI medical students.

We focus on: (1) self-assessed confidence in performing medical tasks in English versus Mandarin Chinese, and (2) perceptions of EMI utility for succeeding in clinical assessment administered in the native-language. As a pilot study, our objective is to identify key patterns and themes for a subsequent larger longitudinal study.

## 2 Methods

### 2.1 Participants

Twenty-four fourth-year medical students (12 female, 12 male; mean age 22.9 years) participated from an eligible cohort of 29 (82.8% response rate). All were native Mandarin speakers enrolled in the EMI track and had completed the NCT examination. Written informed consent was obtained, and the study received ethical approval from the MED Institutional Review Board Office at The Chinese University of Hong Kong, Shenzhen (IRB Number: 20250150) (20).

CUHK-Shenzhen was selected for its Hong Kong-derived EMI curriculum in mainland China, geographically diverse student body (spanning Shenzhen, Guangdong, and nationwide), and admissions policies selecting high-scoring Gaokao students with strong English proficiency, thereby isolating EMI pedagogy effects from inadequate language preparation.

All participants completed 3 years of preclinical EMI instruction and 1 year of clinical rotations in Mandarin-speaking hospitals, with no formal medical Mandarin training. The program mirrors other Chinese MBBS programs that use EMI, but serves local students rather than international cohorts.

### 2.2 Instruments

A novel survey instrument was developed for this study through a multi-stage process. First, items were generated based on a review of medical competency frameworks (21), and existing language assessment tools (22), adapted for the Chinese EMI context. Second, three bilingual medical educators reviewed the draft for content validity, relevance, and clarity. The final instrument comprised three sections: Section A (English Medical Competence) with 10 items assessing confidence in performing core medical tasks in English; Section B (Mandarin Chinese Medical Competence) with 10 parallel items assessing confidence in performing identical tasks in Mandarin; and Section C (Perceptions of EMI Utility) with 7 items assessing perceptions of how useful EMI training was for various aspects of the Chinese clinical context. All items used a 5-point Likert scale (1 = Lowest confidence/ability, 5 = Highest confidence/ability). Internal consistency was good, with Cronbach’s *α* = 0.899 for Section A, 0.869 for Section B, and 0.815 for Section C. The survey was administered in person using paper formats within 1 week of NCT completion. All materials were presented in English, the language of instruction, with no translation provided as participants were proficient English speakers enrolled in the EMI program.

To complement the survey findings and explore the cognitive dimensions of linguistic transfer in greater depth, semi-structured interviews were conducted with a subset of participants (*n* = 7). Interviewees were purposively selected to represent a range of confidence levels. The interview protocol explored: (1) experiences navigating English instruction and Mandarin clinical practice, (2) cognitive processes during clinical reasoning and language switching, (3) perceptions of EMI utility for NCT performance, and (4) suggestions for curriculum improvement. Interviews were conducted with seven students (female *n* = 4, male *n* = 3). English was used for questioning, although some students were permitted to use Mandarin for certain words to reduce cognitive load. The interviews, which lasted 13–22 min, were audio-recorded, transcribed verbatim, and translated where necessary. Translation of Chinese vocabulary was performed using Microsoft Translator (Chinese to English) and then rechecked by two bilingual staff members, who compared the translations with the original transcripts and resolved any discrepancies through discussion.

### 2.3 Data analysis

All analyses were performed in R (version 4.3.0), and figures were generated using the ggplot2 package. Analyses were descriptive, consistent with the pilot study’s aims.

#### 2.3.1 Quantitative analysis

For each of the 10 parallel tasks, a paired difference score (Chinese confidence—English confidence) was computed. The magnitude of each gap was expressed as Cohen’s *d*. To evaluate whether participants demonstrated statistically significant differences between their English and Chinese confidence scores across the 10 medical tasks, we conducted paired *t*-tests for each task. A two-sided *p* < 0.05 was considered statistically significant. Response frequencies for the 7 utility items were summarized to identify trends of high (score 4–5) versus low (score 1–2) agreement.

#### 2.3.2 Qualitative analysis

Interview transcripts were analyzed using thematic analysis. Two researchers independently reviewed the transcripts to identify recurring patterns and themes related to language transfer, cognitive burden, utility perceptions, and curriculum suggestions. Themes were discussed and refined iteratively until consensus was reached. Illustrative quotations were selected to represent the range of participant experiences and to provide explanatory depth for quantitative findings.

## 3 Results

The results are presented in two parts. The first part reports quantitative findings from the survey, including NCT performance, language confidence, and perceptions of EMI utility. The second part presents qualitative interview data that explains these findings.

**Figure 2:**
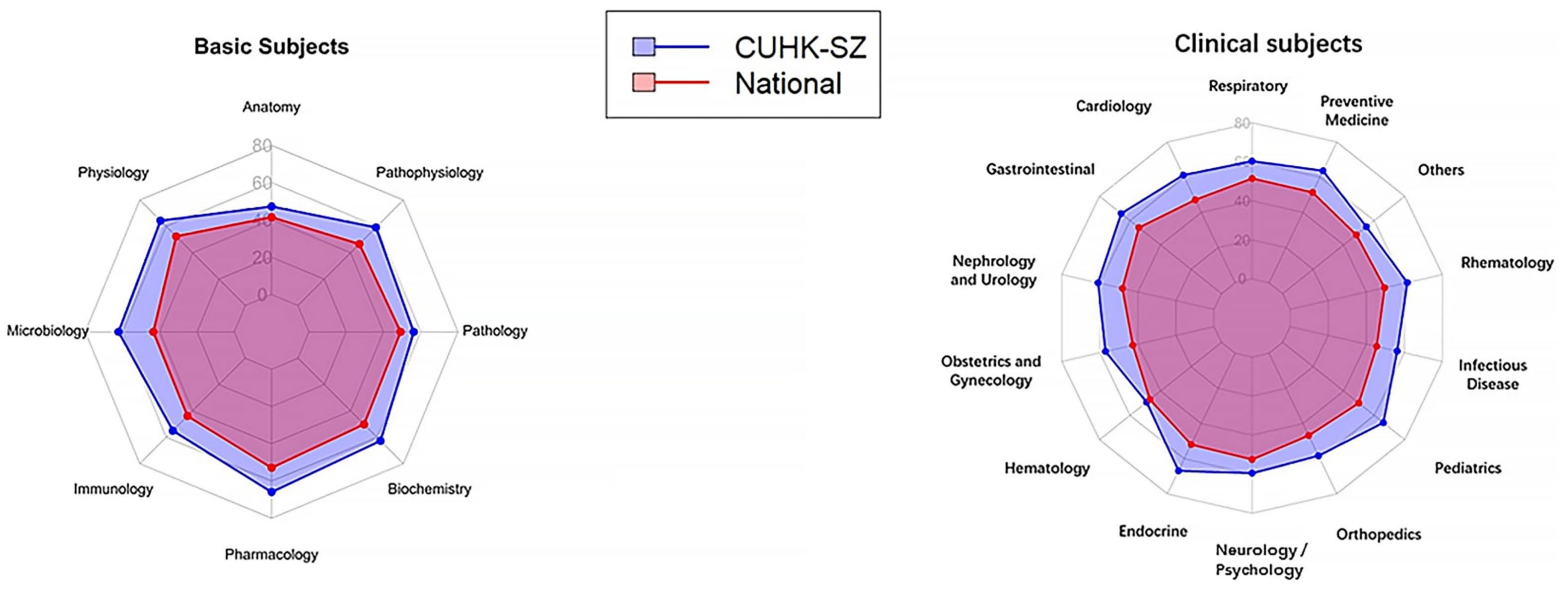
Comparison of the National Competency Test (NCT) correctness in each domain between CUHK-SZ and the national average level.

The EMI cohort delivered outstanding performance on the Chinese-language National Competency Test (NCT). According to the official institutional report, all students achieved a 100% pass rate on both the written (Basic Medical Knowledge) and clinical skills (OSCE) components. The average comparison of the cohort with the national level is presented in Figure 2. The cohort’s mean written exam score was 232.17 (against a 180-point passing threshold), placing it in the 99.21st percentile nationally. Their mean OSCE score was 92.50. Thirteen students achieved scores within the national top 10% on the written component, while none scored in the bottom 10%. Performance was consistently strong across all assessed domains. In the Basic Medical Knowledge component, the cohort demonstrated superior mastery in every module compared to national averages: Basic Medical Sciences (73.74% vs. 59.75%), Clinical Medicine (77.82% vs. 63.99%), Medical Humanities (75.69% vs. 66.19%), and Preventive Medicine (79.66% vs. 64.25%). Within the clinical skills (OSCE) stations, students excelled notably in history-taking (Station 2: 96.03% mastery) and clinical procedures (Station 5: 94.31% mastery), with high performance maintained across all six stations.

**Figure 3:**
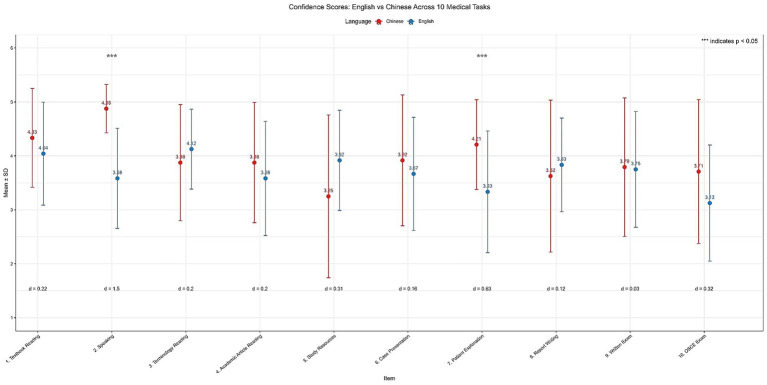
Comparison of self-reported confidence in 10 medical tasks between Chinese and English.

The confidence level difference across 10 medical tasks between English and Chinese domains was illustrated in Figure 3. Students reported slightly higher overall confidence performing medical tasks in Chinese than in English, with a small effect size (Chinese Mean = 3.95, SD = 0.84; English Mean = 3.70, SD = 0.76; Cohen’s *d* = 0.20; *p* = 0.34). Students reported varying levels of confidence across medical tasks in Chinese and English (Figure 3). Confidence was significantly higher in Chinese for two productive, communication-oriented tasks, as indicated in the figure by the triple asterisks. The largest gap was observed for general speaking ability (*p* < 0.001; *d* = 1.50), indicating substantially greater confidence speaking in Chinese. A medium-sized and statistically significant gap also appeared for explaining information to patients (*p* = 0.005; *d* = 0.63), again favoring Chinese. For all other tasks, confidence differences were small and not statistically significant. Reading-related tasks, including textbook reading (*d* = 0.22), terminology reading (*d* = 0.20), and academic article reading (*d* = 0.20), showed minimal differences between the two languages. Similar small effects were found for study resources (*d* = 0.31), case presentation (*d* = 0.16), report writing (*d* = 0.12), written exams (*d* = 0.03), and OSCE performance (*d* = 0.32).

Details can be found in Supplementary Table 1.

A clear pattern emerged across domains: confidence gaps were most pronounced for productive skills requiring real-time verbal communication, while receptive tasks involving comprehension showed much smaller differences. Students reported comparable confidence in reading and understanding medical material in both languages but felt notably less prepared to use English for spontaneous clinical communication.

Interview data provided insight into the mechanisms underlying this confidence gap. Students consistently described the cognitive burden of translating between English learning and Mandarin practice. One participant explained: “I find this process difficult, as sometimes I don’t even know the Chinese translation of the terminology” (P3). Another noted: “It is hard, because we learn in English without an English clinical environment” (P7). This translation burden helps explain the significant confidence gap in clinical speaking (*d* = 1.50), as students must simultaneously recall clinical content and retrieve appropriate Mandarin terminology under time pressure. Students linked their low speaking confidence to insufficient practice opportunities: “They speak too little. Need more speaking practice” (P1); “Lack of opportunities (to) practice verbal communication skills” (P3). These qualitative accounts reinforce the survey finding that productive skills lag behind receptive abilities.

Pearson and Spearman correlation analyses showed a moderate positive association between Chinese-language OSCE confidence (Item B10) and actual clinical skills OSCE scores. Pearson’s r was 0.46 (*p* = 0.03), and Spearman’s r was 0.42 (*p* = 0.04), both indicating statistically significant correlations.

**Figure 4:**
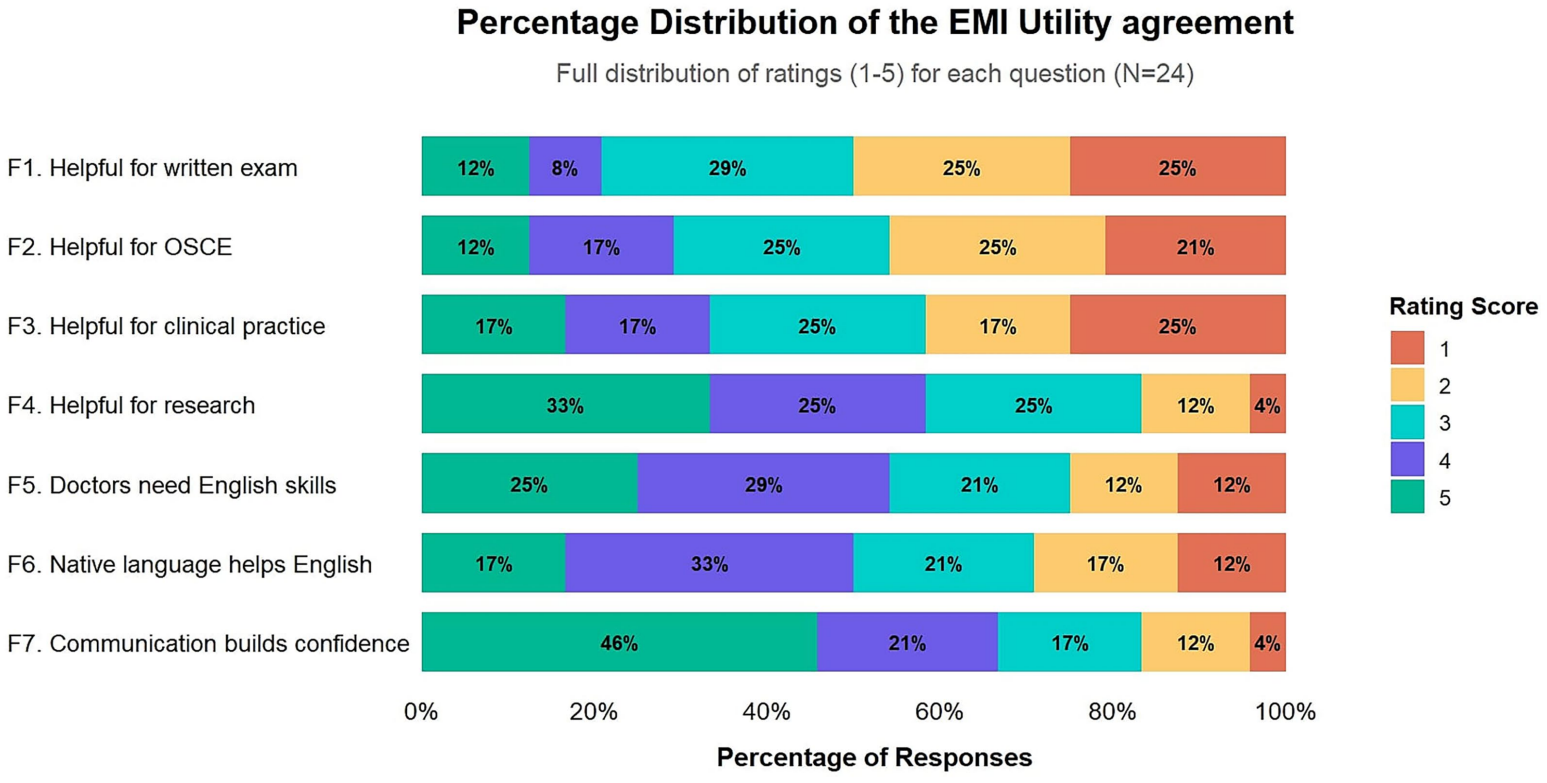
Stacked bar of the percentage distribution of the EMI utility agreement.

Participants’ perceptions of EMI utility were nuanced and context-dependent (Figure 4; Table 1). A strong consensus valued EMI for enhancing global competencies and research preparedness, with 87.5% agreement that practicing communication in English increased confidence in English skills and 79.2% agreement that EMI training would benefit a future research career. In contrast, a majority expressed low agreement that their EMI background was helpful for either the written exam (58.3%) or the OSCE (54.2%) components of the NCT.

**Table 1:** Summary and interpretation of the perception item score interval.

Perception item	Mean (SD)	% High (4–5)	% Low (1–2)	Interpretation
Helpful for the written exam	2.58 (1.32)	20.8%	50%	Low utility
Helpful for the OSCE	2.75 (1.33)	29.2%	45.8%	Low utility
Helpful for clinical practice	3.83 (1.43)	33.3%	41.7%	Moderate utility
Helpful for research	3.71 (1.20)	58.3%	16.7%	High utility
Doctors need English	3.42 (1.35)	54.2%	25.0%	Moderate agreement
Native language helps English skills	3.25 (1.29)	50.0%	29.2%	Moderate agreement
Communication builds confidence	3.92 (1.25)	66.7%	16.7%	High agreement

This performance-utility paradox is notable: despite reporting limited perceived utility of EMI for the NCT (written exam *M* = 2.29; OSCE *M* = 2.38), the cohort demonstrated exceptional academic proficiency. Correlational analysis revealed that success on the Chinese OSCE was not linked to confidence in performing tasks in Chinese (*r* = −0.01) but showed a weak-to-moderate positive correlation with confidence in English (*r* = 0.31), suggesting that foundational knowledge acquired through EMI may be associated with exam success.

Interview data provided explanatory depth. Students overwhelmingly attributed their NCT success to self-study rather than EMI instruction: “I would attribute greater than 70% to my self-study” (P3); *“Self-study in Chinese 80–90%”* (P1). However, some recognized conceptual transfer: “The basic philosophy is the same. Language is not the problem” (P5). Students identified assessment style differences as a key factor in this disconnect: “The assessment style: NCT requires reciting more than reasoning” (P7). These perceptions suggest students view EMI as fostering reasoning while viewing the NCT as testing recall—a distinction that obscures EMI’s foundational role.

### 3.1 Correlation between self-reported confidence and examination performance

Pearson and Spearman correlation analyses were conducted to examine the relationship between students’ self-reported confidence in completing an OSCE medical examination in Chinese (Item B10) and their actual examination performance (as measured by clinical skills OSCE scores). The results showed a moderate positive association between Chinese-language OSCE confidence and the clinical skills OSCE score. Pearson’s r was 0.46 (*p* = 0.03), and Spearman’s r was 0.42 (*p* = 0.04), both indicating statistically significant correlations.

These findings suggest that students who report greater confidence in completing an OSCE in Chinese tend to perform better in the clinical skills examination.

### 3.2 Perceptions of EMI training utility

Based on the results of the survey, participants’ perceptions regarding the utility of EMI training were nuanced and demonstrated a clear, context-dependent trend. Based on the rating interval distribution results shown in Figure 4 and further interpretation in Table 1. A strong consensus valued EMI for enhancing global competencies and research preparedness, while simultaneously perceiving its direct utility for the native-language licensure examination as limited.

This dichotomy is evident in the specific responses. The highest levels of agreement were associated with items stating that practicing communication in English increased confidence in English skills (87.5% agreement) and that EMI training would be beneficial for a future research career (79.2% agreement). In stark contrast, a majority of participants expressed low agreement that their EMI background was helpful for either the written exam (58.3%) or the OSCE (54.2%) components of the Chinese National Medical Licensing Examination (NCT), a perception that stands in notable contrast to their cohort’s exceptional second-place national ranking. Interview data corroborated this finding, with students consistently attributing their NCT success to self-study rather than EMI instruction.

## 4 Discussion

This pilot study provides preliminary insights into a critical phase of EMI medical education: the transition from English-language learning to native-language licensure and practice. The data reveal two prominent patterns that warrant further investigation in a larger, controlled study.

First, the data reveal a significant confidence gap in students’ ability to perform clinical duties in English versus Chinese, highlighting a critical divergence between the medium of academic acquisition and the medium of professional performance. This aligns with the linguistic threshold hypothesis, which stipulates that a foundational level of second-language proficiency is necessary for the effective transfer of subject-specific knowledge (23). Furthermore, it resonates with cognitive load theory; the additional processing demands of operating in a less-automatic academic language may impede the fluid retrieval and application of knowledge in complex, time-pressured clinical scenarios (16, 24).

It has been asserted that language barriers in foreign-language medical education pose considerable challenges (25); however, they may not constitute an insurmountable obstacle to success. The exceptional NCT performance of this cohort provides a compelling counterpoint. It suggests that the EMI model, rather than hindering learning, equipped students with a transferable and deep understanding of medicine. Several factors may explain this success. First, instruction in English provided access to a superior breadth and depth of learning resources, facilitating comprehensive subject understanding (2). Second, engaging with complex concepts in two linguistic systems likely reinforced learning through dual-coding and comparative analysis, enhancing overall topic comprehension (26, 27). Third, the requirement to later apply this knowledge in Mandarin for exams may have forced a deeper level of processing, moving students beyond memorization of English terms to a genuine grasp of concepts that could be articulated in another language (4). Therefore, teaching in the common global language of medicine does not inherently create a barrier; it can establish a strong foundational knowledge that is readily applicable in one’s native language.

In such an environment, therefore, students may develop strong compensatory strategies, such as self-study and peer collaboration, which allow them to master the material and excel. Consequently, these barriers may function as a demanding filter that can foster resilience and innovative learning. Thus, students’ awareness of a language-skill gap should not be interpreted as a manifestation of the Dunning-Kruger effect, but rather as a critical step toward overcoming it (28). This insight represents a pivotal metacognitive shift, where students acknowledge that transitioning from academic comprehension to fluent clinical communication requires dedicated, deliberate practice in the target language.

Our results reflect the powerful influence of the “hidden curriculum” (29), the pervasive, unofficial process of professional socialization. Here, the hidden curriculum implicitly communicates that true medical competence is demonstrated in Mandarin for clinical practice and licensure, a message that directly shapes student priorities and can overshadow the formal curriculum’s explicit valuation of global English proficiency.

Second, the findings reveal a distinct hierarchy in how students value their EMI education. Its utility is perceived most strongly for facilitating global academic engagement and research, a perception that directly aligns with the strategic objectives of implementing EMI (30). This is pragmatically substantiated by the reality of the global medical discourse, where English serves as the lingua franca for approximately 86.5% of clinical research publications (2).

However, they perceive minimal direct benefit for passing the Mandarin-language NCT examination. This pragmatic view reflects the examination’s linguistic format but raises a crucial pedagogical question. If students do not see a clear pathway from EMI learning to success in a native-language assessment, it may create motivational conflicts. This could encourage strategic learning behaviors that prioritize parallel, exam-focused study in Chinese, a finding that echoes research on “compartmentalization” in bilingual education (31).

This is critical, as passing the NMLE is mandatory for medical licensure in China. The perceived utility of an EMI program in relation to this examination is therefore a significant factor for both domestic students and aspiring international students who intend to practice clinically in China.

## 5 Conclusion

The study identifies a core dilemma within EMI medical education in China: while students recognize the value of English for global competence and research and achieve strong outcomes on national exams, they perceive a significant disconnect between acquiring knowledge in English and applying it within the Chinese clinical and assessment context. The cohort’s exceptional examination performance challenges this perception and may provide empirical support for the argument that learning medicine in English, the field’s global lingua franca, does not create a barrier to practicing in one’s native language. On the contrary, the cognitive engagement with two languages and access to English resources can enhance deep learning, creating a transferable knowledge base that facilitates success in native-language licensure and practice (33). Thus, while a Hong Kong-style EMI curriculum can sufficiently prepare students to pass the NCT examination, it still presents substantial challenges for developing professional confidence. The significant confidence gap, especially in speaking, highlights that EMI affects professional confidence more than examination performance. Ultimately, the success of EMI depends not merely on English instruction but on proactively bridging this linguistic divide to ensure knowledge transfer into local practice. Addressing this challenge requires solutions adapted to China’s specific demographic and linguistic realities.

### 5.1 Implications for practice and curriculum design

Based on these findings, EMI programs may aim to evolve beyond the model of simple language substitution in teaching. The data highlights several critical areas where intentional curricular redesign is needed to bridge the identified gaps.

First, implementing elements of an integrated bilingual pedagogy is essential. Curricula ought to include explicit “bridging” activities, including the creation of dual-language glossaries, the execution of comparative terminology analysis, and the utilization of structured translation exercises. These activities are designed to actively connect knowledge acquired in English with its application in the native-language clinical context. Such activities should be framed not as remedial support, but as exercises that leverage the cognitive advantages of bilingual learning to solidify conceptual mastery and demonstrate the direct transferability of knowledge from the English-medium classroom to Chinese-language practice (32). Proactive interventions, beginning well before clinical rotations, are needed to systematically prepare students for the linguistic and procedural demands of local clinical practice and licensing assessments. This approach may facilitate a more confident and effective transition for students.

Second, the substantial confidence gap in productive skills necessitates targeted clinical communication training. Programs should implement structured practice in clinical English, including standardized patient interactions and role-playing scenarios focused on history-taking, diagnosis explanation, and patient counseling. This practice is important even within a predominantly Chinese-speaking clinical environment.

Third, to address the observed performance-utility paradox, transparent curriculum alignment is crucial. Educators must clearly map and demonstrate for students how the competencies and knowledge gained through EMI instruction directly correspond to the requirements of high-stakes, native-language licensure examinations like the NMLE. This involves clearly demonstrating for students how the proven strengths of EMI, including access to global resources and the analytical depth gained from comparing medical terminology, are foundational to the skills required for the high-stakes licensure examination.

### 5.2 Limitations and future directions

The primary limitations of this pilot study are its small, single-institution sample and cross-sectional design, which limit generalizability and preclude causal inference. The reliance on self-reported confidence, as opposed to objective measures of language performance, constitutes a further constraint. However, the integration of qualitative interviews with seven participants adds explanatory depth, illuminating the cognitive processes and perceptions underlying quantitative patterns.

These findings provide a foundation for more definitive research. A larger-scale follow-up study is warranted. To address the present limitations, a longitudinal, cohort study is planned. Ideally, such a study would include data from students enrolled in other EMI clinical medicine programs in China. This study will track students from program entry through the NCT, using validated surveys administered before and after the examination to quantify changes in confidence and perceived utility. The qualitative component will be expanded to explore student perceptions in greater detail across multiple institutions. This multi-method design will allow for a more comprehensive investigation of linguistic transfer within EMI medical education.

## Data Availability

The raw data supporting the conclusions of this article will be made available by the authors, without undue reservation.
