# Supplementary material for: Bridging language gaps: a study of linguistic transfer perceptions among Chinese medical students in an English-medium instruction program
Source: Front Med (Lausanne). 2026 May 4;13:1798909. doi: 10.3389/fmed.2026.1798909 (PMC13180615; doi:10.3389/fmed.2026.1798909)
Supplement: Supplementary file 1 [file Data_Sheet_1.PDF]

Supplementary Material

1 Supplementary Data

NOT APPLICABLE

2 Supplementary Figures and Tables

2.1 Supplementary Figures

NOT APPLICABLE

2.2 Supplementary Tables

2.2.1 Appendix- Supplementary Table 1

	Confidence in English		Confidence in Chinese			
	mean	SD	mean	SD	p_value	Cohen's d
Textbook Reading	4.04	0.95	4.33	0.92	0.30	0.22
**Speaking**	**3.58**	**0.93**	**4.88**	**0.45**	**<0.001**	**1.50**
Terminology Reading	4.13	0.74	3.88	1.08	0.33	0.20
Academic Article Reading	3.58	1.06	3.88	1.12	0.34	0.20
Study Resources	3.92	0.93	3.25	1.51	0.14	0.31
Case Presentation	3.67	1.05	3.92	1.21	0.44	0.16
**Patient Explanation**	**3.33**	**1.13**	**4.21**	**0.83**	**0.005**	**0.63**
Report Writing	3.83	0.87	3.63	1.41	0.56	0.12
Written Exam	3.75	1.07	3.79	1.28	0.89	0.03
OSCE Exam	3.13	1.08	3.71	1.33	0.13	0.32
Overall	3.70	0.76	3.95	0.84	0.34	0.20

2.1.2 Survey questionnaires form

香港中文大學（深圳）
The Chinese University of Hong Kong, Shenzhen
醫學院

Student ID No.: ______________________________
Name: ______________________________
Year of Study: ______________________________

What is your native language?:
Mandarin Chinese / English / Both / Other ______________________________

Before NCT Exam

Instructions: Please rate your confidence/ability on a scale of 1 (Lowest) to 5 (Highest).

Section A: English Language Confidence

A1. How confident are you with reading English medical textbooks or references?
1 2 3 4 5

A2. How confident do you consider yourself speaking in English?
1 2 3 4 5

A3. How confident are you with reading medical terminology in English?
1 2 3 4 5

A4. How confident are you with reading a medical academic research article in English?
1 2 3 4 5

A5. How often do you use English-language medical resources to study?
1 2 3 4 5

A6. What would you rate your ability to present a medical case in English to colleagues?
1 2 3 4 5

A7. What would you rate your ability to explain medical details to a patient in English?
1 2 3 4 5

A8. What would you rate your ability to write a medical history report in English?
1 2 3 4 5

A9. What would you rate your confidence in completing a written medical exam in English?
1 2 3 4 5

A10. What would you rate your confidence in completing an OSCE medical exam in English?
1 2 3 4 5

Section B: Chinese Language Confidence

B1. How confident are you with reading Chinese medical textbooks or references?
1 2 3 4 5

B2. How confident do you consider yourself speaking in Chinese?
1 2 3 4 5

B3. How confident are you with reading medical terminology in Chinese?
1 2 3 4 5

B4. How confident are you with reading a medical academic research article in Chinese?
1 2 3 4 5

B5. How often do you use Chinese-language medical resources to study?
1 2 3 4 5

B6. What would you rate your ability to present a medical case in Chinese to colleagues?
1 2 3 4 5

B7. What would you rate your ability to explain medical details to a patient in Chinese?
1 2 3 4 5

B8. What would you rate your ability to write a medical history report in Chinese?
1 2 3 4 5

B9. What would you rate your confidence in completing a written medical exam in Chinese?
1 2 3 4 5

B10. What would you rate your confidence in completing an OSCE medical exam in Chinese?
1 2 3 4 5

Section C: Reflection on English Medical Training

C1. How helpful was your English medical training background for the Chinese written exam?
1 2 3 4 5

C2. How helpful was your English medical training background for the Chinese OSCE?
1 2 3 4 5

C3. How helpful do you believe your English medical training background will be for practicing clinical medicine in a Chinese environment?
1 2 3 4 5

c4. How helpful do you believe your English medical training background will be for an academic research career in a Chinese environment?
1 2 3 4 5

C5. How confident do you agree that doctors in non-English-speaking countries should have sufficient medical English skills?

1 2 3 4 5

C6. How confident do you agree that being proficient in medical discussions in your native language helps you more easily develop similar skills in English?

1 2 3 4 5

C7. How confident do you agree that communicating with colleagues in English will increase my confidence in English skills?

1 2 3 4 5
